# ACTIVITY INTENSITY AND HEALTH DECLINES IN OLDER ADULTS WITH PROSTATE CANCER VIA THE OPEN-SOURCE GERI PLATFORM

**DOI:** 10.1093/geroni/igae098.2598

**Published:** 2024-12-31

**Authors:** Nabiel Mir, Yan Che, Russell Szmulewitz, Megan Huisingh-Scheetz

**Affiliations:** University of Chicago Hospitals, Chicago, Illinois, United States; University of Chicago, Chicago, Illinois, United States; University of Chicago, Chicago, Illinois, United States; University of Chicago, Chicago, Illinois, United States

## Abstract

Wearable sensors may facilitate remote monitoring of patient vulnerability during cancer treatment but the associations between the technology measures and clinically meaningful measures have not been established. The Geriatric Remote Initiative (GeRI) is a remote data collection platform that employs a Bangle.js 2 wrist accelerometer alongside a WiFi-enabled Raspberry Pi tablet to collect and transmit free-living physical activity in older adults with cancer. The objective of this analysis was to relate mean activity levels collected in a pilot sample of 10 advanced prostate cancer patients aged ≥65 years over a period of ≥12 weeks to baseline self-rated health and 12-week adverse events. All participants were currently or previously exposed to androgen deprivation therapy. Participants wore the accelerometer weekly for 48-hours; activity indices were recorded and averaged every 15 minutes. Records with at least 12 hours of activity, two or more measurement periods, and ≥50% wear compliance were included. Mean day time (7am-7pm) activity indices were calculated across wear periods. Data from two devices were omitted due to insufficient activity data capture, resulting in 8 evaluable patients. The participant with the lowest mean day time activity (mean activity index=45,803.2) had the lowest baseline self-rated health and was the only participant to develop new high-grade vascular comorbidities at 12 weeks. The mean activity index for the rest of the sample ranged from 80,454.0 to 154,898.3 (mode of five instances of wear, range 3-6 wear instances). Remote monitoring using platforms like GeRI may offer novel methods for detecting vulnerability throughout cancer treatment.

